# FDF-DB: A Database of Traditional Fermented Dairy Foods and Their Associated Microbiota

**DOI:** 10.3390/nu14214581

**Published:** 2022-11-01

**Authors:** Paola Zinno, Francesco Maria Calabrese, Emily Schifano, Paolo Sorino, Raffaella Di Cagno, Marco Gobbetti, Eugenio Parente, Maria De Angelis, Chiara Devirgiliis

**Affiliations:** 1 Research Centre for Food and Nutrition, CREA (Consiglio per la Ricerca in Agricoltura E L’Analisi Dell’Economia Agraria), Via Ardeatina 546, 00178 Rome, Italy; 2 Department of Soil, Plant and Food Science, University of Bari Aldo Moro, Via Giovanni Amendola 165/a, 70126 Bari, Italy; 3 Department of Biology and Biotechnology “C. Darwin”, Sapienza University of Rome, Piazzale Aldo Moro 5, 00185 Rome, Italy; 4 Department of Electrical and Information Engineering, Polytechnic of Bari, 70125 Bari, Italy; 5 Faculty of Science and Technology, Free University of Bolzano, 39100 Bolzano, Italy; 6 Scuola di Scienze Agrarie, Alimentari, Forestali ed Ambientali (SAFE), Università degli Studi della Basilicata, 85100 Potenza, Italy

**Keywords:** fermented dairy food, microbial communities, cheese, yogurt, fermented milk, food database

## Abstract

Background: Fermented foods are attracting increasing interest due to their nutritional and health benefits, including a positive impact on gut microbiota exerted by their associated microbes. However, information relative to traditional fermented dairy products, along with their autochthonous microbiota, is still fragmented and poorly standardized. Therefore, our aim was to collect and aggregate data useful for obtaining a comprehensive overview translated in a classical database interface that can be easily handled by users. Methods: a preliminary inventory was built up by systematically collecting data from publicly available resources for the creation of a list of traditional dairy foods produced worldwide, including additional metadata useful for stratifying, and collapsing subgroups. Results: we developed the Fermented Dairy Food Database (FDF-DB), a feasible resource comprising 1852 traditional dairy foods (cheeses, fermented milks, and yogurt) for which microbial content and other associated metadata such as geographical indication label, country/region of origin, technological aspects were gathered. Conclusions: FDF-DB is a useful and user-friendly resource where taxonomic information and processing production details converge. This resource will be of great aid for researchers, food industries, stakeholders and any user interested in the identification of technological and microbiological features characterizing traditional fermented dairy products.

## 1. Introduction

Fermented dairy products are widely consumed around the world. Due to the recently increased consumer awareness of their strong implications in nutrition and health status, and their positive impact on gut microbiota, fermented foods are rising in popularity and consumption [1,2,3].

The largest percentage of total consumption of dairy products, particularly cheeses, occurs in Europe and North America, where *per capita* consumption is even expected to increase. According to data reported by the OECD-FAO, cheese consumption will also expand in countries where such products are not part of the traditional diet, for example in Southeast Asia, where rising incomes and increasing urbanization have led to changes in eating habits [4].

Fermented dairy products have a long history, and the first evidence of their existence dates to about 8000 years ago, when the first milk animals were domesticated by our ancestors [5]. Over the centuries, the art of cheese making has spread rapidly throughout the world, where different climate conditions, geographical locations, cheese-associated microbiota, and cultural habits resulted in products with distinctive characteristics including a wide variety of flavors, textures, and tastes strongly related to the area of production and local traditions [6,7]. In modern times, production processes in dairy plants are often far removed from domestic procedures; however, in several countries artisanal procedures are applied based on ancient recipes characterized by manual handling of all cheese-making steps. Within this context, the European Union (EU) has promoted three types of quality labels for agricultural products and foodstuffs as part of its food quality policies, with the specific aim of promoting and protecting their unique characteristics, linked to their geographical origin as well as traditional knowledge. These geographical indications imply intellectual property rights for specific agricultural products or foodstuffs, whose qualities are strictly related to the area of production and comprise: Protected Designation of Origin (PDO), Protected Geographical Indication (PGI) and Traditional Specialty Guaranteed (TSG) [8]. In particular, PDO products are those that have the strongest links to the places in which they are made, and every part of the production, processing and preparation process must take place in the specific region; PGI emphasizes the relationship between the specific geographic region and the name of the product, where a particular quality, reputation or other characteristic is essentially attributable to its geographical origin and for most products, at least one of the production stages, processing or preparation takes place in the reference region; Traditional Specialty Guaranteed (TSG) highlights the traditional aspects, such as the way the product is made or its composition, without it being linked to a specific geographical area (https://ec.europa.eu/info/food-farming-fisheries/food-safety-and-quality/certification/quality-labels/quality-schemes-explained_en, accessed on 15 September 2022).

In the United Kingdom (UK), Geographical Indication (GI) schemes protect registered product names when they are sold in Great Britain (England, Scotland, and Wales), Northern Ireland and the EU. All product names protected in the EU from the 31 of December 2020 following successful applications to the EU GI schemes are also protected under the UK and EU GI schemes (https://www.gov.uk/guidance/protected-geographical-food-and-drink-names-uk-gi-schemes, accessed on 15 September 2022).

Recently, a growing interest in fermented foods has emerged, related to the benefits that some of the microorganisms provide to human health, especially their contribution to a healthy gut microbiome and their potential role as probiotics [9,10,11]. Fermented products are a complex category of foods, which have been recently described by an expert panel of the International Scientific Association for Probiotics and Prebiotics (ISAPP) as “foods made through desired microbial growth and enzymatic conversions of food components”. Regardless of the presence of living microbes, all foods and beverages obtained through fermentation, within the food matrix at the time of consumption were included in the above-mentioned category [12]. Because of their content in live bacteria and yeast, dairy products can be considered as one of the most important sources of foodborne microbes ingested upon consumption. These microorganisms represent a complex consortium characterized by a high biodiversity in terms of microbial strains of environmental origin [13]. The increasing of metataxonomic sequencing at low prices magnifies the possibility to easily gather and analyze taxa relative abundances in a growing number of fermented dairy foods [14].

However, information relative to traditional fermented dairy products, along with their autochthonous microbiota, is still fragmented and poorly standardized. This is principally due to the lack of raw data and consequently to the impossibility of analyzing them in a pool using specific federated meta-analysis methods [15]. Therefore, it is attractive and sound to collect and aggregate data to obtain a comprehensive database easily available to users. To this purpose, we developed the Fermented Dairy Food Database (FDF-DB), a user-friendly resource including a high number of traditional dairy food products worldwide distributed, inspected for their microbial taxa content (at phylum and species level), and some associated metadata including geographical indication label, country/region of origin, as well as technological aspects (milk source, milk treatment, ripening). With respect to other existing databases, the advantages of FDF-DB are the simple interface and the relational database system, in which the “product name” represents the primary key to usefully link all the other related tables.

## 2. Materials and Methods

### 2.1. Search Strategy and Data Collection

The total number of cheeses and milk-based beverages was collected systematically from publicly available sources (specific internet sites, institutional portals, and databases) between April 2020 and March 2022, by using and combining the search terms “cheese” or “dairy product” or “fermented milk” and the name of the country through the usage of Boolean operators. Traditional products were selected, while industrial products were excluded from the analysis. All the retrieved records were listed and inventoried using csv files processed by Unix bash shell. As reported in Table 1, the inventory also included other information and metadata.

Subsequently, a systematic literature search for peer-reviewed research articles was carried out both on PubMed and Scopus databases, using each product name as a search item. The total number of retrieved results was reported (Table 2). Among such references, a number of articles reporting the food microbiota composition through molecular techniques was indicated and selected to extrapolate the categories of microbial phyla, genera, and species, for each product. This information was included in the database.

### 2.2. Database Structure

**Figure 1:**
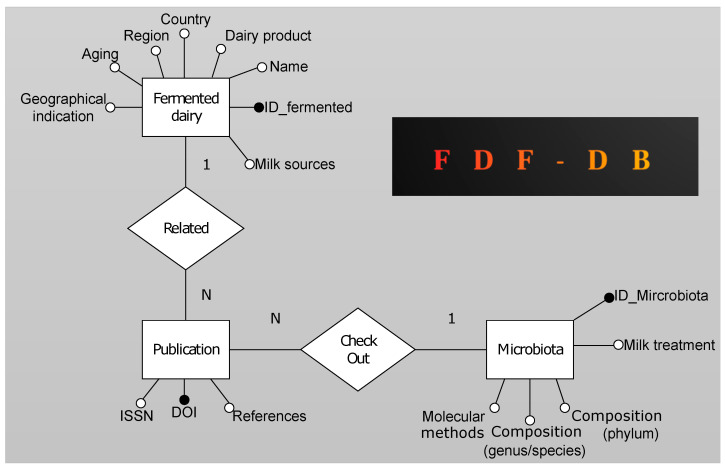
Entity relationship diagram of Fermented diary database. The diagram represents relationships among the stored entities.

Results are presented in a tabular format, with each record depicting a dairy product, that contains the respective reference, DOI number and microbial composition. The complete set of gathered data was used to create the online database accessible at https://quintadb.pro/dccTW7 (accessed on 15 September 2022) by using the pair “FDF-DB” and “intimic” as login and password, respectively, for the basic HTTP authentication. Figure 1 shows the entity relationship diagram of the fermented dairy database. The diagram consists of three entities Fermented, Publication and Microbiota. Between the entities Fermented-Publication and Publication-Microbiota, there is an association of type 1 to N, which indicates that the publication table will contain the foreign key (black dots) of the Fermented and Microbiota entity. The foreign keys are essential for joining entities to obtain a single table (using a join clause) containing all the attributes of the three entities. This element structure allows query runs.

The implemented structure is reported in Figure 1.

## 3. Results and Discussion

### 3.1. Collection and Inventory of Dairy Products

The main purpose of this work was to provide a comprehensive picture of microbiomes associated with fermented foods in terms of dairy product classification, production methods and molecular identified taxa. Given their popularity and worldwide diffusion, we focused our attention on traditional dairy products containing live microbes. To this aim, an inventory was initially built up by collecting a list of dairy foods produced in different countries all over the world. The search strategy, described in Materials and Methods, retrieved a total of 1852 dairy food items, comprising cheeses, fermented milks, and yogurts (Supplementary Table S1). Products were divided according to the country of production, and there is the possibility that the same product was associated with different countries. Additional metadata related to each product were also included in the inventory and used to categorize the foods within the database (see Table 1 for the description of classification terms).

**Figure 2:**
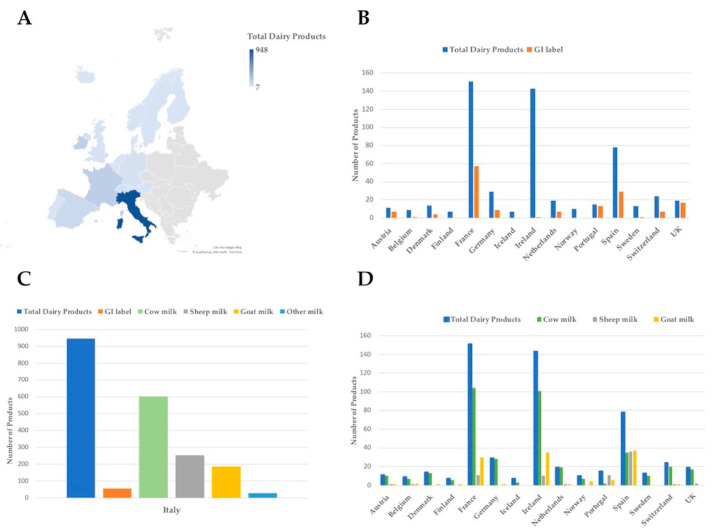
Distribution of dairy products within Western European countries: Panel (**A**) map of Western Europe showing the distribution of traditional dairy products for each country. The number of products is represented by a color gradient, spanning from 7 (Finland and Iceland) to 948 (Italy); Panel (**B**) number of total (blue) and GI labelled (orange) dairy products for each country; Panel (**C**) number of total dairy products (blue), GI labelled (orange), and number of those derived from cow (green), sheep (grey), goat (yellow) or other milk sources, for Italy; and Panel (**D**) number of total dairy products (blue), and number of those derived from cow (green), sheep (grey) or goat (yellow) milk, for each country.

The overall distribution of dairy products throughout different continents/geographical regions is represented in Table 2. More specifically, 94 products were identified from Africa, Asia, and the Middle East, 78 products from the Americas, while 183 and 1497 were associated with Eastern and Western Europe, respectively (Table 2 and Table 3, Supplementary Tables S2–S4). Due to the historical use and knowledge of the cheese-making processes, it was not surprising that we found the substantial majority of traditional fermented dairy products belonging to Western European countries, in particular to Italy and France, followed by Ireland and Spain (Table 3), in line with previous evidence reported by others [16,17]. For these reasons, we gave particular emphasis to the European traditional dairy foods collected and included in the database. Within European countries, a geographical indication label was available for a subset of products with different relative proportions, except for Finland, Iceland and Norway which did not include GI-labelled products (Table 3, Supplementary Table S2). A graphical representation of the distribution of total and GI-labelled dairy products within Western Europe is reported in Figure 2. The GI label represents an added value and is an interesting flag for both producers, who aim at differentiating their products in international markets, and thereby improve their competitiveness and profitability; and consumers, who look for foodstuffs with specific, identifiable characteristics, particularly those linked to their geographical origin and their production method [18,19]. A bibliometric study of the three EU food quality certification schemes, namely PDO, PGI and TSG, revealed that Southern European countries, especially Italy, Spain and Portugal, show the greatest number of registrations in these certification schemes, along with the highest number of published articles. Moreover, concerning products, cheese appeared to be the most analyzed [8].

The most-represented type of milk used a as source was cow milk, while sheep and goat milk were less popular overall, except for in Portugal, Spain (Table 3, Figure 1), and Greece, where they were prevalent (Supplementary Table S2). In general, in Eastern Europe, cow and sheep milk sources were almost equivalent (Supplementary Table S2). Because of the close connection with the microbial content purportedly being higher in fresh and short-ripened cheeses with respect to ripened ones, we also collected information relative to product aging when available. We defined as “fresh” as the unripened products, while the terms “short-ripened” and “ripened” referred to cheeses subjected to a ripening time that we arbitrarily set as shorter than 6 months or longer than 6 months, respectively. Indeed, ripening period can last from weeks to years [20,21] and no universal method of classification exists. Overall, most of the listed products were short-ripened (Table 2), with a strong prevalence in Western Europe (Table 3), while fresh dairy products were very represented in Asia, the Middle East, and Eastern Europe (Table 2 and Supplementary Tables S2 and S3), reflecting the yogurt, and fermented dairy beverage traditions within these countries [13,22]. Among the research articles retrieved by querying PubMed and Scopus databases using each product name (alone or followed by “cheese” or “milk” term) as search items, a small proportion described food microbiota composition, and only a few of them applied molecular techniques (Table 2 and Supplementary Tables S2–S4). Indeed, several articles were dated before 2005, when the use of classical microbiological approaches was more common. All articles providing information on microbiota composition were selected to extract the categories of microbial taxa for each product, to be included in the database. In particular, the data related to microbial composition were provided in terms of phyla, genera and species as reported by each article, and were not connected to sequencing data. Moreover, the list of molecular methods used to analyze microbial composition was indicated according to the corresponding articles. The advent of high throughput *omics* techniques, such as 16S rRNA gene based Next Generation Sequencing (NGS) and whole genome shotgun metagenomics, allowed for a more comprehensive picture of the composition of complex microbial communities, retrieving information at the species or genus level, also including under-represented taxa [23]. Specifically, the application of such methods and the development of sophisticated bioinformatics tools capable of handling issues related to the dairy sector (the so called cheesomics) have provided deeper insights into the composition and potential functionality of cheese microbiota compared to the information provided by culture-dependent approaches [24]. Emphasis was therefore given to the different methodological approaches employed for microbiota analysis. The articles reporting results related to food microbiota composition were also collected and included in the database, along with DOI, when available.

### 3.2. Database Content and Description

#### Query System

Starting from the above-described inventory, we developed the FDF-DB (Fermented Dairy Food Database) comprising a total of 1852 entries and which has been provided with a user-friendly graphical interface that includes the possibility of search and subset the data. The online FDF-DB is accessible online at the following short url with anti-bot protection link: https://quintadb.pro/dccTW7 (Poland, accessed on 15th of September 2022).

The structure of the database is outlined in Figure 1 above and described in Section 2.

Briefly, the list of included fields accounted for: flag number, country, dairy product classification, product name, geographical Indication label, milk sources, product aging, region, references, ISSN, references, DOI, microbiota composition, molecular methods, and milk treatment. The database embedded functions to allow public users to query the data by using various inclusion/exclusion searching criteria allowing also for partial word-matching searches.

More precisely, the available query functions for specific fields included perfect or partial text pattern searches and the possibility of finding empty fields. The main non-redundant field was represented by the “product name” as the main key of the database table.

Among the available functions there was also the option to group the listed hits by using a specific field.

The option to search a simple database and collapse results under specific stratified variables will be of great aid in merging and collapsing results based on specific features, i.e., geographic areas. In this view, users can search, select, and download data subsets for further elaborations.

Moreover, the “country” field has been used to place the number of fermented dairy products with respect to each nation by using the application map powered by Google. By clicking on the nation flag, it is possible to access the associated information and download the information as single items.

Other databases have been developed by other researchers. One large biological resource for dairy foods is represented by the FoodMicrobionet database [25], built up following the need to gather and harmonize data obtained by 16S rRNA gene targeted high-throughput sequencing, and subsequently updated over the years [26,27]. With respect to FoodMicrobionet, the herein adopted strategy relies on the concept of collecting and analyzing downloaded raw data from different available datasets (worldwide shared) and running them under the same bioinformatics pipelines with the same parameters.

FermFooDb is a recently developed database focused on bioactive peptides derived from a wide range of fermented foods including milk, cheese, yogurt, wheat, and rice, with the aim of selecting novel food ingredients for the creation of commercial by the food industries [28].

Omics Database of Fermentative Microbes (ODFM) is an integrated data management system containing information on genome, metagenome, metataxonome, metatranscriptome, and metabolome of fermentative microorganisms associated with various foods. Within this database, food sources are expressed as samples and include kimchi, fermented seafoods, soybean paste, solar salt, vinegar, and alcohol fermentation starter [29]. The Probiotic Database collects probiotics from several fermented foods, including traditional dairy products, providing biological information for use in humans, animals, and plants [30].

In addition, the European Food Safety Authority (EFSA) released FoodEx2, a hierarchical food classification and description system useful for describing food in data collections across different food safety domains [31].

To the best of our knowledge, this is the first database that has collected thousands of traditional dairy products, and has resulted from the merge between product properties and microbial taxonomic information as derived from culture-dependent, molecular techniques and 16S rRNA gene targeted high-throughput sequencing obtained from published data.

Efforts were spent to connect fundamental technological, microbiological, and geographical aspects related to traditional dairy products. A comprehensive view of such features is useful considering the need for a better characterization of traditional fermented dairy products, especially in terms of their linked microbial patterns. Unlike nutrients such as proteins, carbohydrates, and fats, the microbial contents of foods are not available in dietary composition databases. Indeed, the suggestion to include fermented foods in national dietary guidelines is emerging, based on the potential contribution of the health-promoting features of autochthonous live microbes, when present [12,32,33]. Quantification of microbial content of specific foods, along with their potential colonization ability, could help in defining a recommended daily intake of microbes [34], as recently reported in a study conducted on the US population [35].

## 4. Conclusions

FDF-DB is a useful resource where taxonomic information and processing production details related to traditional fermented dairy products converge. A friendly interface allows for an easy consultation helpful not only for researchers, but also for food industries and stakeholders in the selection and identification of technological and microbiological features characterizing the traditional dairy products collected in the database. This will allow for: (i) the identification of existing gaps of information related to specific microbial communities characterizing a product of interest that could be filled by future research; (ii) the identification of promising microbial species/taxa which can be further characterized or employed in industrial applications; and (iii) the possibility of merging the microbial taxa derived from different dairy foods. Although a considerable number of traditional dairy products from all over the world have been collected from the available literature, the database is not exhaustive, and it will be periodically updated with the most recent information. Indeed, we plan to: expand the database with other products and include eventual additional metadata that could be useful for further connecting technological, microbiological, and nutritional properties. These actions will also envisage contributions from other research fields.

The present database represents a valuable resource for scientific, industrial and consumer communities.

## Figures and Tables

**Table 1:** Classification terms through which the different fermented dairy products were categorized in the database. Each searched item is a dedicated column in the database.

Classification Term	Description
Product name	The common or usual name of the food; some products are produced in different countries with the same name and therefore appear more than once in the database.
Country/Region	The country where the product is manufactured. When available, region name was added in a separate field.
Geographical indication label	Quality label referred as Protected Designation of Origin (PDO), Protected Geographical Indication (PGI) and Traditional Specialty Guaranteed (TSG), as defined by the European Union for traditional foods.
Milk sources	Description of milk animal source (goat milk, sheep milk, cow milk, etc.).
Product aging	Ripening of cheese, when available: fresh (unripened); short ripened (<6 months); ripened (>6 months).
Articles describing microbiota composition	Number of articles reporting results related to dairy product microbiota composition, highlighting the applied molecular techniques, when available.
References	List of articles reporting results related to food microbiota composition. When available, the digital object identifier (DOI) was added in a separate column.
Other references	List of additional references from other sources
Microbiota composition	List of bacterial and fungal species associated to each product, according to the related articles. Phylum and genus and species (or higher taxa when genus and species were not available) were specified in separate columns.
Molecular methods	List of molecular techniques used to analyze microbial composition, according to the corresponding articles.
Milk treatment	Eventual heat treatment applied to milk used to obtain the dairy product: raw (no heat treatment) or pasteurized.

**Table 2:** Overall distribution of identified traditional dairy products, along with their characteristics and related articles, within continents or geographical regions.

Continent/Geographical Region	Total Dairy Products	Geographical Indication Label	Cow Milk *	Sheep Milk *	Goat Milk *	Other Milk *	Fresh *	Short Ripened *	Ripened *	Articles in PubMed or Scopus	Articles Describing Microbiota Composition
Africa, Asia, Middle East	94	n.a.	52	14	16	19	74	12	5	1255	44
Americas	78	n.a.	70	10	9	0	32	34	10	1090	8
Eastern Europe	183	44	83	84	65	8	82	71	18	3722	75
Western Europe	1497	209	986	328	308	34	192	1038	434	5971	176
TOTAL	1852	253	1191	436	398	61	380	1155	467	12,038	303

* The sum of the items Cow milk, Sheep milk, Goat milk and Other milk outnumbers the corresponding Total dairy products, since in some cases the same product can be obtained by using different milk sources. The same applies also to Fresh, Short ripened and Ripened items.

**Table 3:** Distribution of the identified traditional dairy products, along with their characteristics and related articles, within Western Europe countries.

Country	Total Dairy Products	Geographical Indication Label	Cow Milk *	Sheep Milk *	Goat Milk *	Other Milk *	Fresh *	Short Ripened *	Ripened *	Articles in PubMed or Scopus	Articles Describing Microbiota Composition
Austria	11	7	10	1	1	0	0	8	4	24	3
Belgium	9	1	7	1	2	0	0	8	1	127	3
Denmark	14	4	13	0	1	0	2	12	0	122	2
Finland	7	0	6	0	1	1	3	4	0	63	2
France	151	57	104	11	30	3	4	121	20	734	40
Germany	29	9	28	0	1	0	3	22	4	261	3
Iceland	7	0	3	0	0	0	6	1	0	65	0
Ireland	143	1	101	10	35	0	3	88	40	5	5
Italy	948	56	604	254	188	28	149	667	309	2738	80
Netherlands	19	7	19	1	1	1	2	14	7	657	3
Norway	10	0	7	0	4	0	5	4	3	61	2
Portugal	15	13	2	11	6	0	0	15	0	14	3
Spain	78	29	35	36	37	0	10	36	26	684	28
Sweden	13	1	10	0	0	1	3	6	3	26	0
Switzerland	24	7	20	1	1	0	1	17	11	309	0
UK ^a^	19	17	17	2	0	0	1	15	6	81	2
TOTAL	1497	209	986	328	308	34	192	1038	434	5971	176

* The sum of the items Cow milk, Sheep milk, Goat milk and Other milk outnumbers the corresponding Total dairy products, since in some cases the same product can be obtained by using different milk sources. The same applies also to Fresh, Short ripened and Ripened items. ^a^ UK: United Kingdom (Galles, Scotland, England).

## Data Availability

All data included in the paper are part of the online database feasibly accessible for the basic HTTP authentication at https://quintadb.pro/dccTW7 (accessed on 27 October 2022) using “FDF-DB” and “intimic” as login and password, respectively.

## Supplementary Materials

The following supporting information can be downloaded at: https://www.mdpi.com/article/10.3390/nu14214581/s1, Table S1: List of products included in the database; Table S2: Distribution of the identified traditional dairy products, along with their characteristics and related articles, within Eastern Europe, Balcanic and Russian countries; Table S3: Distribution of the identified traditional dairy products, along with their characteristics and related articles, within Africa, Asia and Middle East countries; Table S4: Distribution of the identified traditional dairy products, along with their characteristics and related articles, within American countries.
