# Supplementary material for: FDF-DB: A Database of Traditional Fermented Dairy Foods and Their Associated Microbiota
Source: Nutrients. 2022 Nov 1;14(21):4581. doi: 10.3390/nu14214581 (PMC9658602; doi:10.3390/nu14214581)
Supplement: Supplementary file 1 [file nutrients-14-04581-s001.zip › Table S2.pdf]

Table S2. Distribution of the identified traditional dairy products, along with their characteristics and related articles, within Eastern Europe, Balcanic and Russian countries

Country	Total dairy products	Geographical indication label	Cow milk*	Sheep milk*	Goat milk*	Other milk*	Fresh*	Short ripened*	Ripened*	Articles in PubMed or Scopus	Articles describing microbiota composition
Albania	6	n.a.	1	3	3	0	3	3	1	2	0
Macedonia, Serbia, Bosnia-Erzegovina	10	n.a.	8	6	6	1	1	6	1	18	1
Bulgaria	6	0	4	4	3	1	3	1	2	44	1
Croatia	1	0	0	1	0	0	0	1	0	0	0
Cyprus	4	1	1	2	3	0	2	3	0	98	2
Czech Republic	5	3	4	1	0	0	0	5	0	0	0
Estonia	1	0	1	0	0	0	0	1	0	0	0
Greece	31	22	5	26	25	0	13	20	2	836	20
Hungary	11	0	5	2	2	0	6	5	0	3	0
Kosovo	1	n.a.	1	1	0	0	0	1	0	0	0
Latvia	1	0	n.d.	n.d.	n.d.	n.d.	1	0	0	0	0
Lithuania	4	3	2	0	0	0	2	1	1	1	0
Malta	1	0	0	1	0	0	1	1	0	0	0
Moldova	4	n.a.	1	4	1	0	3	2	0	9	0
Poland	7	5	2	3	0	0	2	0	0	2	0
Romania	9	1	5	5	2	0	5	2	2	35	0

Russia, Ucraine, Belarus	18	n.a.	9	2	2	0	11	2	0	31	0
Slovakia	10	5	5	4	0	0	4	3	0	17	6
Slovenia	5	4	4	1	1	0	1	4	0	4	0
Armenia, Azerbaigian, Georgia	9	n.a.	5	3	3	4	7	1	1	2131	44
Turkey, Turkestan	39	n.a.	20	15	14	2	16	9	8	491	5
Kurdistan	1	n.a.	n.d.	n.d.	n.d.	n.d.	1	0	0	0	0
**TOTAL**	**183**	**44**	**83**	**84**	**65**	**8**	**82**	**71**	**18**	**3722**	**79**

* The sum of the items Cow milk, Sheep milk, Goat milk and Other milk outnumbers the corresponding Total dairy products, since in some cases the same product can be obtained by using different milk sources. The same applies also to Fresh, Short ripened and Ripened items.
