# Supplementary material for: FDF-DB: A Database of Traditional Fermented Dairy Foods and Their Associated Microbiota
Source: Nutrients. 2022 Nov 1;14(21):4581. doi: 10.3390/nu14214581 (PMC9658602; doi:10.3390/nu14214581)
Supplement: Supplementary file 1 [file nutrients-14-04581-s001.zip › Table S3.pdf]

Table S3. Distribution of the identified traditional dairy products, along with their characteristics and related articles, within Africa, Asia and Middle East countries

Country	Total dairy products	Geographical indication label	Cow milk*	Sheep milk*	Goat milk*	Other milk*	Fresh*	Short ripened*	Ripened*	Articles in PubMed or Scopus	Articles describing microbiota composition
Afghanistan	2	n.a.	0	0	0	1	2	0	0	1	0
Algeria	1	n.a.	0	0	1	0	1	0	0	0	0
Bangladesh	1	n.a.	1	0	0	1	0	1	0	25	0
Benin	1	n.a.	1	0	0	0	0	0	1	0	0
Burkina Faso	1	n.a.	1	0	0	0	1	0	0	9	1
Burundi	1	n.a.	n.a	n.a	n.a	n.a.	1	0	0	1	0
Cameroon	1	n.a.	n.a	n.a	n.a	n.a.	1	0	0	1	1
Central Asia	6	n.a.	3	1	0	3	6	0	0	44	0
China	5	n.a.	3	0	1	1	3	2	0	28	2
Egypt	8	n.a.	4	1	1	4	4	2	3	149	4
Ethiopia	4	n.a.	1	0	0	0	4	0	0	8	0
India	7	n.a.	4	0	2	2	4	1	1	407	5
Indonesia	1	n.a.	n.a	n.a	n.a	n.a	1	0	0	1	0
Iran	7	n.a.	2	2	1	0	7	0	0	147	3
Japan	1	n.a.	1	0	0	0	0	1	0	0	0
Jordan	1	n.a.	0	1	1	0	1	0	0	0	0
Kenya	5	n.a.	4	1	1	2	5	0	0	26	5
Lebanon	3	n.a.	2	0	0	0	3	0	0	95	1

Middle East	17	n.a.	9	7	6	0	9	5	0	155	1
Mongolia	5	n.a.	2	1	1	2	5	0	0	63	10
Nepal	2	n.a.	2	0	0	2	2	0	0	11	0
Nigeria	2	n.a.	1	0	0	0	2	0	0	7	2
Pakistan	2	n.a.	1	0	1	1	2	0	0	1	1
Rwanda	1	n.a.	1	0	0	0	1	0	0	1	0
South Africa	2	n.a.	2	0	0	0	2	0	0	33	2
Sudan	1	n.a.	1	0	0	0	1	0	0	6	3
Tanzania	2	n.a.	2	0	0	0	2	0	0	0	0
Zambia	2	n.a.	2	0	0	0	2	0	0	9	1
Zimbabwe	2	n.a.	2	0	0	0	2	0	0	27	2
**TOTAL**	**94**	**0**	**52**	**14**	**16**	**19**	**74**	**12**	**5**	**1255**	**44**

* The sum of the items Cow milk, Sheep milk, Goat milk and Other milk outnumbers the corresponding Total dairy products, since in some cases the same product can be obtained by using different milk sources. The same applies also to Fresh, Short ripened and Ripened items.
